# Efficacy and Safety of Isatuximab, Carfilzomib, and Dexamethasone (IsaKd) in Multiple Myeloma Patients at the First Relapse After Autologous Stem Cell Transplantation and Lenalidomide Maintenance: Results from the Multicenter, Real-Life AENEID Study

**DOI:** 10.3390/ph18040595

**Published:** 2025-04-19

**Authors:** Nicola Sgherza, Olga Battisti, Paola Curci, Concetta Conticello, Salvatore Palmieri, Daniele Derudas, Candida Germano, Enrica Antonia Martino, Giuseppe Mele, Roberta Della Pepa, Francesca Fazio, Anna Mele, Bernardo Rossini, Giulia Palazzo, Daniela Roccotelli, Simona Rasola, Maria Teresa Petrucci, Domenico Pastore, Giuseppe Tarantini, Fabrizio Pane, Massimo Gentile, Francesco Di Raimondo, Emanuela Resta, Pellegrino Musto

**Affiliations:** 1 Hematology and Stem Cell Transplantation Unit, AOUC Policlinico, 70124 Bari, Italy; nicolasgherza@libero.it (N.S.); olgabattisti@gmail.com (O.B.); paolacurci@tiscali.it (P.C.); 2 Division of Hematology and Stem Cell Transplantation, Policlinico “G. Rodolico-San Marco”, 95123 Catania, Italy; ettaconticello@gmail.com (C.C.); diraimon@unict.it (F.D.R.); 3 Hematology Unit, Ospedale Cardarelli, 80131 Napoli, Italy; salvatore.palmieri@aocardarelli.it; 4 Department of Hematology, Businco Hospital, 09121 Cagliari, Italy; derudas74@gmail.com; 5 Hematology Unit, “Dimiccoli” Hospital, 70051 Barletta, Italy; candida.germano@aslbat.it (C.G.); giuseppetarantini0@gmail.com (G.T.); 6 Department of Onco-Hematology, Hematology Unit, Azienda Ospedaliera Annunziata, 87100 Cosenza, Italy; enricaantoniamartino@gmail.com (E.A.M.); massimo.gentile@unical.it (M.G.); 7 Hematology Unit, “Perrino” Hospital, 72100 Brindisi, Italy; giuseppemele2007@gmail.com (G.M.); domenico.pastore0@gmail.com (D.P.); 8 Hematology—Department of Clinical Medicine and Surgery, University Hospital “Federico II”, 80131 Napoli, Italy; robertadellapepa@gmail.com (R.D.P.); fabrizio.pane@unina.it (F.P.); 9 Department of Translational and Precision Medicine Hematology, Sapienza University, 00185 Roma, Italy; fazio@bce.uniroma1.it (F.F.); petrucci@bce.uniroma1.it (M.T.P.); 10 Hematology Unit, “Cardinale Panico” Hospital, 70039 Tricase, Italy; annamele@hotmail.com; 11 Hematology and Cell Therapy Unit, IRCCS Istituto Tumori “Giovanni Paolo II” Bari, 70124 Bari, Italy; bernardorossini@gmail.com; 12 Haematology Unit, Ospedale G. Moscati, 74010 Taranto, Italy; giuliapalazzo@libero.it; 13 Department of Hematology and Bone Marrow Transplant, IRCSS “Casa Sollievo Della Sofferenza”, 71013 Foggia, Italy; d.roccotelli@gmail.com; 14 Department of Precision and Regenerative Medicine and Ionian Area, “Aldo Moro” University School of Medicine, 70121 Bari, Italy; simona.rasola@gmail.com; 15 Department of Pharmacy, Health and Nutritional Science, University of Calabria, 87036 Rende, Italy; 16 Department of General Surgery and Medical-Surgical Specialties, Hematology Section, University of Catania, 95124 Catania, Italy; 17 Department of Clinical and Molecular Medicine, Sapienza University, 00185 Roma, Italy; restaemanuela@gmail.com

**Keywords:** first-relapsed multiple myeloma, second-line therapy, isatuximab–carfilzomib–dexamethasone, autologous stem cell transplantation, lenalidomide resistance

## Abstract

**Background**: In the randomized, phase-3 IKEMA trial, the triplet isatuximab, carfilzomib, and dexamethasone (IsaKd) demonstrated superior clinical benefit compared to those of carfilzomib and dexamethasone alone in patients with relapsed/refractory multiple myeloma after 1–3 prior treatments. **Methods**: Our real-world, AENEID study aimed to evaluate the efficacy and safety of IsaKd in patients who relapsed after frontline lenalidomide treatment, poorly represented in the IKEMA trial. Specifically, in the present multicenter analysis, we enrolled eighty-two patients who received, between April 2022 and September 2024 and outside of clinical trials, at least one cycle of IsaKd as a second-line treatment at the first relapse after induction therapy, autologous stem cell transplantation (ASCT), and lenalidomide maintenance. **Results**: After a median follow-up time of 12.9 months (range, 1–77), the overall response rate, at least a very good partial response rate, and median progression-free survival time were 79.3%, 56.1%, and 24.4 months, respectively. This slightly lower performance compared to that in the IKEMA study may be attributed to the well-known poor prognostic impact of lenalidomide refractoriness (len-R), developed by all our patients during maintenance therapy, and to a higher proportion of patients with extramedullary disease present in our series, which was identified as the only factor significantly affecting the PFS in multivariable analysis. The median overall survival was not reached, as in the pivotal trial, while the 1-year survival probability was 85.1%. Regarding the safety profile, our findings were consistent with those of the IKEMA trial, with no new safety signals reported. **Conclusions**: These real-world data support the use of IsaKd as a valuable option for len-R MM patients relapsing after the first-line therapy, including ASCT and lenalidomide maintenance.

## 1. Introduction

Patients with refractory or relapsed multiple myeloma (MM) after first-line treatments, including lenalidomide—either in combination with dexamethasone alone (Rd), as a part of triplets, such as bortezomib–lenalidomide–dexamethasone (VRd) or daratumumab–lenalidomide–dexamethasone (DRd), or as a maintenance therapy following autologous stem cell transplantation (ASCT)—represent a growing and clinically relevant population [1,2,3]. In this context, most combination treatments currently available in daily practice as second-line therapies were approved based on pivotal trials evaluating triplet regimens incorporating daratumumab or isatuximab (in patients not refractory to anti-CD38 antibodies), pomalidomide, and bortezomib or carfilzomib [4,5,6,7,8,9]. However, in most of these studies, detailed data on the clinical outcomes of patients who are truly refractory to lenalidomide (len-R) after only one prior line of therapy remain limited.

Promising results come from the prospective, open-label, phase-3 IKEMA trial (clinicaltrials.gov identifier: NCT03275285) [8,9], in which 302 patients with refractory/relapsed MM (RRMM) were randomly assigned to receive isatuximab (Isa) in combination with carfilzomib (K) and dexamethasone (d) (IsaKd) (179 patients) versus Kd (123 patients) after a median of two prior lines of therapy (range: 1–3). Isatuximab is an IgG1 monoclonal antibody that targets a specific epitope of CD38, inducing myeloma cell death through multiple mechanisms, including antibody-dependent cell-mediated cytotoxicity, complement-mediated cytotoxicity, and direct apoptosis [10,11]. Carfilzomib, a second-generation proteasome inhibitor, irreversibly binds the active sites of the 20S proteasome, as well as the core component within the 26S proteasome; by selectively inhibiting these proteasomes, carfilzomib delays proliferation and induces apoptosis in malignant plasma cells [12].

In the most recent update of the IKEMA trial [9], after a median follow-up time of 44 months, the median progression-free survival (mPFS) time was 35.7 months in the intention-to-treat population treated with IsaKd, with 72 patients (40%) considered as lenalidomide exposed and 57 patients (32%) as len-R. However, the number of patients who had progressed on frontline lenalidomide was, indeed, very small (*n* = 8), and a specific mPFS for this subgroup could not be evaluated.

Therefore, to further contribute to this clinically relevant issue, the AENEID study (A rEtrospective, observatioNal, multicEnter study of Isatuximab, car-filzomib and Dexamethasone as second line treatment in multiple myeloma patients relapsed/refractory after an initial therapy including lenalidomide) aimed to evaluate the efficacy and safety of IsaKd in this specific subset of patients, who were underrepresented in the IKEMA trial.

## 2. Results

### 2.1. Patients

Eighty-two patients (median age: 62 years), treated, at the first relapse, with IsaKd between April 2022 and September 2024, after induction therapy, ASCT, and lenalidomide maintenance, were included in this study. At the data cut-off point, the median follow-up time was 12.9 months (range: 1–77.1). The baseline characteristics of the patients are summarized in Table 1.

Most of the patients (86.6%) received bortezomib–thalidomide–dexamethasone as induction therapy and a single ASCT (53.7%). The median duration of the lenalidomide maintenance was 20 months (range: 1–60). Guideline-based dose reduction of lenalidomide was opted for in 31 patients (37.8%) mainly because of hematological toxicities. Sixty-seven patients (81.7%) showed symptomatic relapse; 15 (18.3%), a biochemical relapse. At the start of the IsaKd, thirty-eight patients (46.3%) were in ISS stage I; twenty-three (28.1%), in stage II; twelve (14.6%), in stage III; while in nine patients (11%), ISS was not available. Cytogenetic data were available for 50 patients (61%), 13 of whom were classified at high risk (26%), considering the same criteria of the IKEMA trial (the presence of del(17p), t(4;14), or t(14;16)), while in 15 patients (30%), chromosome 1q abnormalities were observed. Extramedullary disease (EMD) was reported in 16 cases (19.5%). Twenty-seven patients (32.9%) showed cardiac comorbidities (Table 1) before starting IsaKd therapy.

### 2.2. Efficacy

**Figure 1:**
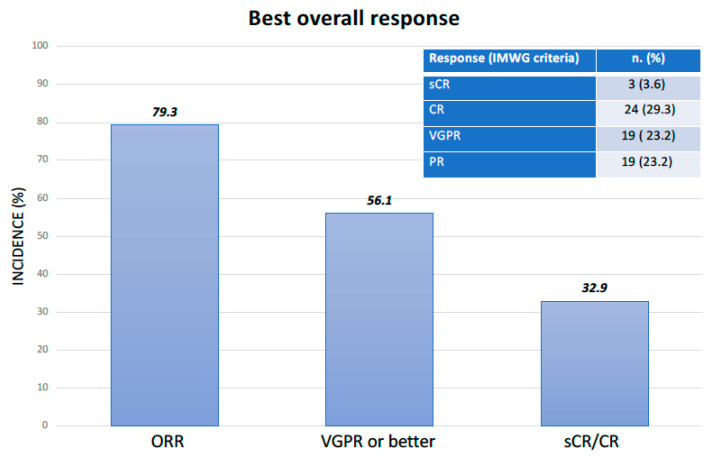
Treatment efficacy.

At the last follow-up time, the median number of IsaKd cycles administered was 7 (range: 1–24). Sixty-five patients (79.3%) achieved at least a partial response (PR) (Figure 1), with a median of three cycles (range: 1–11); more specifically, three patients (3.6%) achieved a stringent complete response (sCR); twenty-four (29.3%), a complete response (CR); nineteen (23.2%), a very good partial response (VGPR); and nineteen (23.2%), a PR.

Moreover, the median time from the initial treatment to the initiation of the second-line IsaKd was 40.5 months (range: 20–105). The median times to the first response and the best response were 2 months (range: 1–4) and 3 months (range: 1–12), respectively.

**Figure 2:**
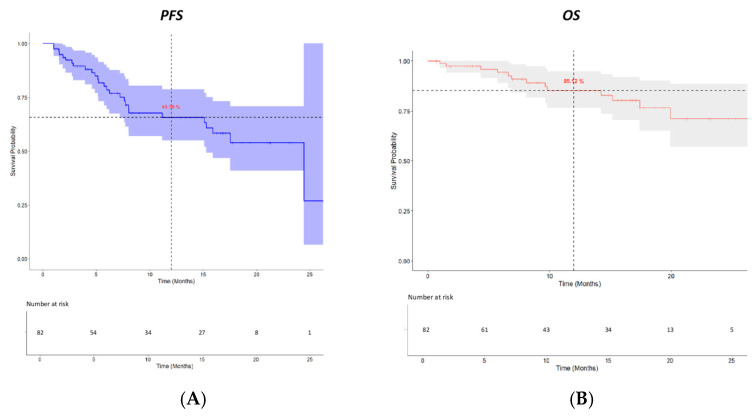
Progression-free survival (**A**) and overall survival (**B**).

After a median follow-up time of 12.9 months, twenty-four (29.3%) patients experienced disease progression or death. The mPFS time was 24.4 months (95% CI: 15.27-NA), and the 1-year PFS probability rate was 65.76% (Figure 2A).

No differences in terms of PFS were observed considering the age (<62 vs. ≥62 years; HR = 0.98, 95% CI: 0.93–1.04; *p* = 0.360), the ISS stage (II + III vs. I; HR = 2.15, 95% CI: 0.95–4.87; *p* = 0.062), the duration of maintenance ((≤12 vs. > 12 months; HR = 0.87, 95% CI: 0.29–2.59; *p* = 0.810)/(≤24 vs. > 24 months; HR = 0.77, 95% CI: 0.36–1.66; *p* = 0.510)), the number of previous ASCTs (single vs. tandem; HR = 1.51, 95% CI: 0.7–3.26; *p* = 0.290), the cytogenetic risk (high vs. standard; HR = 0.5, 95% CI: 0.14–1.74, *p* = 0.260) or the 1q21+ status (absent vs. present; HR = 0.5, 95% CI: 0.14–1.78; *p* = 0.282), the type of relapse (clinical vs. laboratory; HR = 0.65, 95% CI: 0.19–2.17; *p* = 0.480), and LDH values (elevated vs. normal; HR = 0.52, 95% CI: 0.24–1.15; *p* = 0.100). Notably, multivariable analysis showed that EMD was the only parameter significantly associated with an inferior PFS (HR = 0.24, 95% CI: 0.11–0.53; *p* = 0.00013).

A total of 28 (34.1%) patients discontinued the IsaKd treatment, primarily because of disease progression (24 patients, 85.7%). Following IsaKd discontinuation, 19 patients (23.2%) received a subsequent treatment. The median time to the next treatment (TTNT) was 26.3 months (95% CI: 17.2-NA), with a 1-year probability rate of requiring retreatment of 67.9%. More specifically, eleven patients (57.9%) received EloPd (elotuzumab, pomalidomide, and dexamethasone), five patients (26.3%) were treated with D-PACE (dexamethasone, cisplatin, doxorubicin, cyclophosphamide, and etoposide), and the remaining three patients received Ixa-Rd (ixazomib, lenalidomide, and dexamethasone), teclistamab, and Pd, respectively. A total of 13 deaths (15.8%) were recorded. The median overall survival (OS) time was not reached (95% CI, NA-NA), and the 1-year OS probability rate was 85.12% (Figure 2B).

### 2.3. Safety

Infusion-related reactions occurred during the first administration of isatuximab in nine patients (10.9%, all grades 1–2) and were promptly resolved in all the cases, with no treatment discontinuation reported. Hematological toxicity included grades 3/4 thrombocytopenia (30.5%), lymphocytopenia (19.5%), neutropenia (17.1%), and anemia (10.9%) (Table 2).

Among the all-grade infectious events, upper respiratory tract infections and pneumonia were each reported in 11 patients (13.4%), while bacteremia occurred in 3.6% of the subjects (*n* = 3). Four cases of SARS-CoV-2 infections were also reported. Cardiac toxicity consisted of grades I and II hypertension (13.4% of the patients). Guideline-based dose reduction of carfilzomib was opted for in 26 patients (31.7%) because of age, hypertension onset, and hematological toxicity, without compromising the treatment efficacy. Overall, the treatment was permanently discontinued in four patients (4.9%) because of infectious complications. Certainly, because this is a retrospective study, some lower-grade toxicities may have been underreported.

## 3. Discussion

Despite the availability of proteasome inhibitors (PIs), immunomodulatory drugs (IMiDs), and anti-CD38 antibodies, which have significantly improved the prognosis of MM patients in recent years, most still experience a pattern of alternating remissions and relapses [13,14], generally with progressively shorter durations of response to each subsequent regimen [15]. Furthermore, relapses within 12 months after the initial therapy with novel agents are associated with worse prognoses. These patients, currently considered to be at “functional” high risk [16,17], represent an unmet clinical need. Lenalidomide, an IMiD having the CBRL (cereblon) protein as the target for its anti-neoplastic activities, represents a key component of most first-line multi-agent regimens, both in transplant-eligible patients, as a part of induction treatment (VRd) and/or in maintenance therapy following ASCT, and in transplant-ineligible MM patients (VRd, Rd, and DRd) [18]. Because lenalidomide is usually continued until disease progression or intolerance, most MM patients become len-R. Therefore, this aspect represents a relevant issue for the choice of appropriate approaches in the context of patients with RRMM disease, particularly at the first relapse. Notably, no phase-3 trials have been exclusively conducted in len-R patients in this setting, and real-world data remain limited [19,20,21,22].

In reviewing the literature data [23], the phase-3 OPTIMISMM [5,6] and phase-2 EMN011 [24] trials (investigating pomalidomide- and dexamethasone-based combinations with bortezomib or carfilzomib, respectively) are among the few that report outcomes in len-R patients after a single prior line of therapy, particularly in terms of mPFS.

Focusing on patient populations comparable to ours (i.e., len-R patients after the first-line ASCT treatment and lenalidomide maintenance), the OPTIMISMM trial (with a median follow-up time of 16.4 months) showed a mPFS time of 22 months and an overall response rate (ORR) of 91.1% with second-line PVd. Regarding the EMN011 trial, with a longer median follow-up time of 40 months, the mPFS time with carfilzomib–pomalidomide–dexamethasone (KPd) was 32 months, while the ORR and ≥VGPR were 92% and 75%, respectively. Overall, the clinical outcome of the patients treated with IsaKd reported in our analysis was slightly better than that reported in the OPTIMISMM trial but inferior to that observed in the EMN011 study.

In real-world settings, a recent single-center report [19] included 138 len-R MM patients who initiated various salvage regimens after the first-line therapy. The results were disappointing: The ORR to the second-line therapy in len-R patients was 53%, the mPFS time was 10.7 months, and the mOS time was 23.8 months. However, in this study, the percentage of len-R patients receiving ASCT as a part of their first-line treatment was only 26%, with the best estimated mPFS time (18.4 months) obtained using a not otherwise specified combination of PI and IMIDs.

Other real-world experiences in len-R patients treated after one prior therapy have reported quite short mPFS times: 11.9 months (in 11 patients treated with elotuzumab–carfilzomib–lenalidomide–dexamethasone at median follow-up time of 28.2 months) [20] and 15 months (in 79 patients treated with daratumumab–bortezomib–dexamethasone at a median follow-up time of 25 months) [21].

Compared to the IKEMA trial [8,9], in our cohort, both the mPFS time and ORR were lower (24.4 vs. 35.7 months and 79.3% vs. 86.6%, respectively), despite IKEMA patients having received a median number of two prior lines of treatment. However, all the patients in our study were len-R, whereas only eight len-R patients after one line of therapy were included in the IKEMA study, and specific mPFS data were not reported because of the small sample size. Moreover, in our cohort, there was a higher percentage of patients relapsing with EMD (19.5%), a poor prognosis population significantly less represented in the IsaKd arm of the IKEMA study (6.7%) [25]. The prognostic impact of EMD was confirmed in multivariable analysis. As in the IKEMA trial [26], mOS was not reached in our cohort.

De Novellis et al. [22] also reported a subgroup analysis of 69 real-world RRMM patients treated with IsaKd as a second-line therapy, including 47 len-R ones (68%), whose outcomes were, unfortunately, not specified. With a median follow-up time of 12 months, the ORR was 88%, and at least VGPR was 66%. The PFS and OS were not reached but were negatively influenced by the high cytogenetic risk, prior daratumumab exposure, the achievement of less than the VGPR, and the advanced R-ISS stage. Moreover, progression within 12–24 months after initiating lenalidomide maintenance negatively affected the PFS, while severe renal impairment impacted the OS. In this study, no difference emerged based on prior ASCT, the presence of EMD, or lenalidomide exposure/refractoriness status.

Finally, the IONA-MM is an ongoing multinational observational study, including patients with RRMM treated with IsaKd in a real-world setting after a median of two prior lines of therapy [27]. Among 129 patients previously treated with lenalidomide, 65 received IsaKd as a second-line therapy, with 33 being len-R at the last prior line. In len-R patients, the ORR was 72.1% (49.2% ≥ VGPR and 23% ≥ CR). In those treated at the second line, the ORR was 75% (54.2% had ≥ VGPR and 25% had ≥ CR). However, in the latest interim analysis, the data on the IsaKd outcomes, specifically in len-R patients after the first-line ASCT and lenalidomide maintenance, were not reported.

Our study includes the largest cohort of patients treated with IsaKd after intensive first-line lenalidomide-containing treatment, showing encouraging, though still not completely satisfying, results in this selected subset of patients. Notably, the triplet IsaKd demonstrated clinical benefit also in high-risk MM patients, as the PFS did not differ according to the ISS stage or cytogenetic risk, apparently overcoming known prognostic parameters, although the limited number of patients analyzed could have influenced this result.

An emerging area of interest is the prognostic role of chromosome 1q abnormalities [28,29], which negative impact on prognosis has been widely investigated in newly diagnosed MM and appears to be equally relevant in RRMM [30]. In our cohort, 15 patients showed chromosome 1q abnormalities. As in the IKEMA subgroup analysis [31], IsaKd appeared to mitigate the adverse prognostic impact of this cytogenetic abnormality, supporting its role in this difficult-to-treat subgroup of patients.

Poor survival outcomes are also associated with early relapse (within 12 months) after ASCT, even in the era of novel drugs [32,33]. Again, and consistent with the post hoc subgroup analysis from the IKEMA trial about the outcomes of patients with early versus late relapsed MM [34], our data showed no PFS differences based on the maintenance duration, reinforcing the efficacy of IsaKd, regardless of the relapse timing.

Randomized comparisons of double versus single ASCTs following three-drug induction have suggested a possible benefit of tandem ASCT in patients with high-risk disease [35,36]. In our cohort, no differences in terms of the PFS were observed between patients who underwent single versus tandem ASCTs, indicating that IsaKd can achieve clinical goals, regardless of the number of prior transplant procedures.

Regarding the safety profile, our data are consistent with those of the IKEMA trial, with no new safety signals observed. Notably, twenty-seven patients (32.9%) showed cardiac comorbidities, including hypertension and atrial fibrillation, prior to initiating IsaKd therapy. These findings support the safety of this triplet regimen, even in these patients typically underrepresented in clinical trials.

## 4. Materials and Methods

### 4.1. Patients

This retrospective study enrolled 82 patients, at 19 Italian hematology centers, who received at least one cycle of IsaKd, from April 2022 to September 2024, as a second-line treatment (therefore, at the first relapse) after induction therapy, ASCT and lenalidomide maintenance, outside of clinical trials. Data, such as age, gender, date of diagnosis, laboratory parameters, treatment history, and date of the last follow-up or death, were extracted from clinical records at the time of the inclusion and updated on an ongoing basis. The study protocol was reviewed and approved by the involved institutional ethics committees, in accordance with the principles of the Declaration of Helsinki.

### 4.2. Treatment

All the patients were treated with IsaKd according to marketing approval [8]. Specifically, patients received isatuximab (10 mg/kg) intravenously (on days 1, 8, 15, and 22 of the first 28-day cycle and days 1 and 15 of subsequent cycles), carfilzomib (20 mg/sqm) intravenously (on days 1 and 2 of cycle 1) and then at 56 mg/sqm (on days 8, 9, 15, and 16 of cycle 1 and days 1, 2, 8, 9, 15, and 16 of subsequent cycles), and dexamethasone (20 mg) was administered intravenously or orally (on days 1, 2, 8, 9, 15, 16, 22, and 23) until disease progression, unacceptable toxicity, or clinical decision. Premedication included diphenhydramine (25–50 mg) or its equivalent, ranitidine (50 mg) or its equivalent, and acetaminophen (650–1000 mg) or its equivalent, administered 30 to 90 min before isatuximab infusion. Dexamethasone was administered prior to isatuximab infusion as a part of both premedication and study treatment. All the patients received antibacterial and antiviral prophylaxis during treatment according to each center’s policy.

### 4.3. Endpoints

The primary endpoints of our analysis were the ORR (at least the PR), PFS, OS, TTNT, and assessment of the safety profile. The treatment response and disease progression were evaluated according to the International Myeloma Working Group (IMWG) criteria [37,38], with the ORR defined as achieving at least a PR and including the VGPR, CR, and sCR. Safety was evaluated using the National Cancer Institute’s Common Terminology Criteria for Adverse Events version 6.0 (CTCAE v6.0).

### 4.4. Statistical Analysis

Kaplan–Meier analysis was employed to comprehensively evaluate the PFS, OS, and TTNT, using data from datasets after cleaning and preprocessing. The PFS, OS, and TTNT were calculated from the initiation of the IsaKd treatment until disease progression, death, or the last follow-up, and the Kaplan–Meier method was used to estimate survival probabilities, including 1-year probabilities for short-term outcome evaluation. Median values (mPFS, mOS, and mTTNT) and 95% confidence intervals (CIs) were estimated. Log-rank tests were applied to compare survival distributions. Additionally, univariable and multiple Cox regression analyses were performed to assess the prognostic impacts of covariates, such as age, ISS stage, duration of maintenance, number of ASCTs, type of relapse, cytogenetic characteristics, LDH levels, chromosome 1 abnormalities, and EMD presence, providing hazard ratios (HRs) with corresponding 95% CIs. Statistical analyses were conducted using R-4.4.1, utilizing the survival, survminer, and dplyr packages, with a significance threshold set at *p* ≤ 0.05.

## 5. Conclusions

The current study has several limitations, mainly because of its retrospective, real-life design, the absence of a control group, a relatively short follow-up period, and the lack of measurable residual disease (MRD) data, which is an emerging issue also in RRMM. Nevertheless, our analysis supports the clinical efficacy and safety of IsaKd as a first-salvage therapy in a specific, and still existing, subset of MM patients who relapse during lenalidomide maintenance following ASCT. This category, which has not been extensively investigated, remains ineligible for more effective therapies, such as CAR-T and bispecific antibodies.

## Figures and Tables

**Table 1:** Main characteristics of the patients at the time of the IsaKd initiation.

**Median Age, Years (Range)**	62 (43–73)
**Sex, *n* (%)**	
Male	42 (51.2)
Female	40 (48.8)
**M-Protein Type, *n* (%)**	
IgG	47 (57.3)
IgA	19 (23.2)
Light chain only	15 (18.3)
Not secernent	1 (1.2)
**Light Chain Type, *n* (%)**	
Kappa	44 (53.7)
Lambda	37 (45.1)
Not secreting	1 (1.2)
**Creatinine Clearance, *n* (%)**	
<60 mL/min	13 (15.8)
≥60 mL/min	69 (84.2)
**LDH, *n* (%)**	
Normal	23 (28)
Elevated	59 (72)
**β** **2-Microglobulin (mg/L), *n* (%)**	
<3.5	44 (53.7)
≥3.5 <5.5	17 (20.7)
>5.5	12 (14.6)
Unknown or missing	9 (11)
**International Staging System (ISS), *n* (%)**	
I	38 (46.3)
II	23 (28.1)
III	12 (14.6)
Not available	9 (11.0)
**Type of Relapse, *n* (%)**	
Laboratory	15 (18.3)
Clinical	67 (81.7)
FISH Analysis *, *n* (%; Considering Data Available for 50 Patients)	
High risk	13 (26)
Standard risk	37 (74)
**1q21 Abnormalities**	15 (30)
Gain(1q)	9 (60)
Amp(1q)	6 (40)
**Extramedullary Disease, *n* (%)**	16 (19.5)
**Induction Treatment Before ASCT, *n* (%)**	
VTd	71 (86.6)
D-VTd	4 (4.9)
PAd	3 (3.7)
VRd	1 (1.2)
VCd	3 (3.6)
**Autologous Stem Cell Transplantation, *n* (%)**	
Single	44 (53.7)
Tandem	38 (46.3)
**Median Duration of Lenalidomide Maintenance, Months (Range)**	20 (1–61)
<12 months of lenalidomide maintenance; patients, *n* (%)	25 (30.5)
<24 months of lenalidomide maintenance; patients, *n* (%)	48 (58.5)
**Cardiac Comorbidities Before Isa-Kdtreatment, *n* (%)**	27 (32.9)
Hypertension, *n* (%)	24 (29.3)
Atrial fibrillation, *n* (%)	3 (3.6)

* High risk was defined as del(17p), t(4;14), or t(14;16); VTd: bortezomib–thalidomide–dexamethasone; D-VTd: daratumumab–bortezomib–thalidomide–dexamethasone; PAd: bortezomib–adriamycin–dexamethasone; VRd: bortezomib–lenalidomide–dexamethasone; VCd: bortezomib–cyclophosphamide–dexamethasone.

**Table 2:** Safety.

**Hematological Toxicity (Grades III and IV), *n* (%)**	
Thrombocytopenia	25 (30.5)
Lymphocytopenia	16 (19.5)
Neutropenia	14 (17.1)
Anemia	9 (10.9)
**Cardiac Toxicity (Grades I and II), *n* (%)**	
Hypertension	11 (13.4)
**Infectious Events (Any Grade), *n* (%)**	
Pneumonia	11 (13.4)
Upper airway infection	11 (13.4)
SARS-CoV-2 infection	4 (4.9)
Sepsis	3 (3.6)
CMV * infection	2 (2.4)
HZV ** infection	1 (1.2)
Conjunctivitis	1 (1.2)
Cellulitis	1 (1.2)

* Cytomegalovirus; ** herpes zoster virus.

## Data Availability

Although these data are not currently publicly available for sharing, requests for sharing can be sent to the corresponding author and will be evaluated on an individual basis. The data will be provided after their deidentification, in compliance with applicable privacy laws, data protection, and requirements for consent and anonymization.
