# Correction: Sgherza et al. Efficacy and Safety of Isatuximab, Carfilzomib, and Dexamethasone (IsaKd) in Multiple Myeloma Patients at the First Relapse After Autologous Stem Cell Transplantation and Lenalidomide Maintenance: Results from the Multicenter, Real-Life AENEID Study. *Pharmaceuticals* 2025, *18*, 595

**DOI:** 10.3390/ph18060858

**Published:** 2025-06-09

**Authors:** Nicola Sgherza, Olga Battisti, Paola Curci, Concetta Conticello, Salvatore Palmieri, Daniele Derudas, Candida Germano, Enrica Antonia Martino, Giuseppe Mele, Roberta Della Pepa, Francesca Fazio, Anna Mele, Bernardo Rossini, Giulia Palazzo, Daniela Roccotelli, Simona Rasola, Maria Teresa Petrucci, Domenico Pastore, Giuseppe Tarantini, Fabrizio Pane, Massimo Gentile, Francesco Di Raimondo, Emanuela Resta, Pellegrino Musto

**Affiliations:** 1 Hematology and Stem Cell Transplantation Unit, AOUC Policlinico, 70124 Bari, Italy; nicolasgherza@libero.it (N.S.); olgabattisti@gmail.com (O.B.); paolacurci@tiscali.it (P.C.); 2 Division of Hematology and Stem Cell Transplantation, Policlinico “G. Rodolico-San Marco”, 95123 Catania, Italy; ettaconticello@gmail.com (C.C.); diraimon@unict.it (F.D.R.); 3 Hematology Unit, Ospedale Cardarelli, 80131 Napoli, Italy; salvatore.palmieri@aocardarelli.it; 4 Department of Hematology, Businco Hospital, 09121 Cagliari, Italy; derudas74@gmail.com; 5 Hematology Unit, “Dimiccoli” Hospital, 70051 Barletta, Italy; candida.germano@aslbat.it (C.G.); giuseppetarantini0@gmail.com (G.T.); 6 Department of Onco-Hematology, Hematology Unit, Azienda Ospedaliera Annunziata, 87100 Cosenza, Italy; enricaantoniamartino@gmail.com (E.A.M.); massimo.gentile@unical.it (M.G.); 7 Hematology Unit, “Perrino” Hospital, 72100 Brindisi, Italy; giuseppemele2007@gmail.com (G.M.); domenico.pastore0@gmail.com (D.P.); 8 Hematology—Department of Clinical Medicine and Surgery, University Hospital “Federico II”, 80131 Napoli, Italy; robertadellapepa@gmail.com (R.D.P.); fabrizio.pane@unina.it (F.P.); 9 Department of Translational and Precision Medicine Hematology, Sapienza University, 00185 Roma, Italy; fazio@bce.uniroma1.it (F.F.); petrucci@bce.uniroma1.it (M.T.P.); 10 Hematology Unit, “Cardinale Panico” Hospital, 70039 Tricase, Italy; annamele@hotmail.com; 11 Hematology and Cell Therapy Unit, IRCCS Istituto Tumori “Giovanni Paolo II” Bari, 70124 Bari, Italy; bernardorossini@gmail.com; 12 Haematology Unit, Ospedale G. Moscati, 74010 Taranto, Italy; giuliapalazzo@libero.it; 13 Department of Hematology and Bone Marrow Transplant, IRCSS “Casa Sollievo Della Sofferenza”, 71013 Foggia, Italy; d.roccotelli@gmail.com; 14 Department of Precision and Regenerative Medicine and Ionian Area, “Aldo Moro” University School of Medicine, 70121 Bari, Italy; simona.rasola@gmail.com; 15 Department of Pharmacy, Health and Nutritional Science, University of Calabria, 87036 Rende, Italy; 16 Department of General Surgery and Medical-Surgical Specialties, Hematology Section, University of Catania, 95124 Catania, Italy; 17 Department of Clinical and Molecular Medicine, Sapienza University, 00185 Roma, Italy; restaemanuela@gmail.com

## Error in Surname

In the original publication [1], there surname of author Roberta Della Pepa should be Della Pepa.

## Error in Table

In the original publication [1], there was a mistake in Table 1 as published. The number of patients with normal and elevated LDH is 59 (72) and 23 (28), respectively (instead of 23 (28) and 59 (72)). The authors state that the scientific conclusions are unaffected. This correction was approved by the Academic Editor. The original publication has also been updated.
